# A Web-Based and Mobile Intervention Program Using a Spaced Education Approach for Workplace Mental Health Literacy: Cluster Randomized Controlled Trial

**DOI:** 10.2196/51791

**Published:** 2024-04-23

**Authors:** Lawrence T Lam, Mary K P Lam

**Affiliations:** 1 Faculty of Medicine, Macau University of Science and Technology, Macau, China; 2 Faculty of Medicine and Health, The University of Sydney, Sydney, Australia; 3 STEM College, RMIT University, Melbourne, Australia

**Keywords:** mHealth, web-based intervention, mental health literacy, psychoeducation, randomized controlled trial, workplace, performance, worker, intervention, digital health, mental wellness, promote, well-being, mobile health, technology

## Abstract

**Background:**

Workplace mental health is an important global health concern.

**Objectives:**

This unblinded, phase-III, wait-listed cluster randomized controlled trial aimed to examine the effectiveness of a mobile health (mHealth) psychoeducation program using a spaced education approach on mental health literacy (MHL) in the workplace. The main interest of this paper was the immediate and 3-month medium-term effect of the program on the MHL of workers. The purposely built mHealth platform was also evaluated as a health-related app.

**Methods:**

The mHealth platform was designed using the principle of spaced education as a psychoeducation intervention program, with various modules of web-based and mobile materials presented to the participant in a progressive manner. Short quizzes at the end of each module ensured adequate learning, and successful completion qualified the learner to progress to the next level. The trial recruited 456 employees of specific industries with high levels of work-related stress. Participants who were nested in different offices or units were allocated into the intervention and wait-listed control groups using a block randomization process, with the office or unit as the cluster. A separate sample of 70 individual raters were used for the evaluation of the mHealth platform. The Australian National MHL and Stigma Survey and the Mobile Apps Rating Scale were completed through a web-based self-reported survey to assess MHL and evaluate the app. The trial and follow-up data were analyzed by a generalized linear latent and mixed model with adjustments for the clustering effect of work sites and repeated measures.

**Results:**

Of the 456 participants in the trial, 236 (51.8%) responded to the follow-up survey. Most MHL outcomes obtained significant results immediately after the intervention and across time. After adjusting for the clustering effect, the postintervention weighted mean scores were significantly higher in the intervention group than the control group for correct recognition of a mental health problem, help seeking, and stigmatization by 0.2 (SE 0.1; *P*=.003), 0.9 (SE 0.2; *P*<.001), and 1.8 (SE 0.4; *P*<.001), respectively. After adjusting for the clustering effect, significant differences across time were found in help-seeking intention (*P*=.01), stigmatization (*P*<.001), and social distancing (*P*<.001). The evaluation of the mHealth program resulted in average scores of the 4 major domains ranging from 3.8 to 4.2, with engagement having the lowest score.

**Conclusions:**

The mHealth psychoeducation intervention program using this platform had immediate and 3-month medium-term effects of retaining and improving MHL. The platform was evaluated to have satisfactory performance in terms of functionality, aesthetics, information content, and utility in enhancing MHL. It is anticipated that ongoing development in digital health will provide great benefits in improving the mental health of the global population.

## Introduction

Mental ill health has long been recognized as an important global health problem [1]. The workplace has also been identified as an important venue for preventing mental health problems and for promoting mental wellness [2,3]. Preventive programs designed as a workplace strategy could provide benefits to workers in terms of early identification and intervention of mental health problems, as well as promoting mental well-being [4]. One specific approach is to enhance workers’ mental health literacy (MHL). MHL was defined as “knowledge and beliefs about mental disorders which aid their recognition, management or prevention” by Jorm [5]. In this construct, there are different aspects, including the ability to recognize specific disorders, knowledge about mental health, attitudes toward help seeking and stigmatization, and social distancing from people with mental health problems.

In response to the urgent need for a well-designed workplace mental health intervention program in Hong Kong, a group of researchers developed the mobile health (mHealth) web-based and mobile Workplace Mental Health Literacy (WPMHL) project with funding support from the Hong Kong government [6]. The program consists of 2 main components: psychoeducation modules for the enhancement of workers’ MHL and a work environment scan addressing more structural issues in the workplace [6].

**Figure 1.**
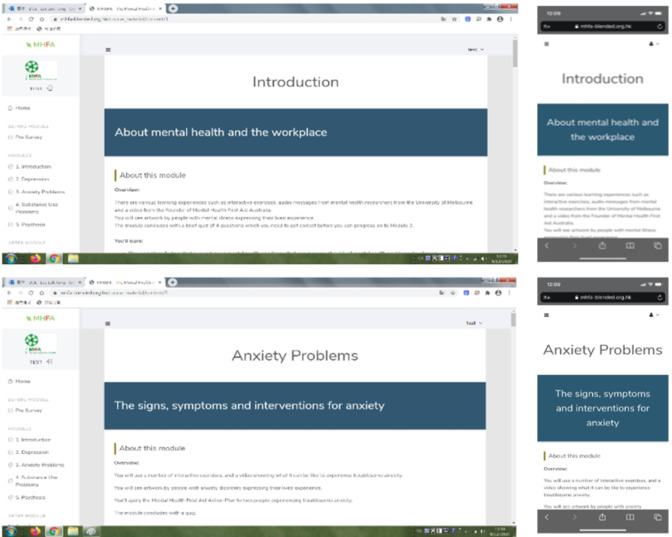
Different versions of the main interface: as a website for PCs (left) and as a downloadable app for tablets and smartphones (right). MHFA: mental health first aid.

The psychoeducation modules of the program incorporated elements of mental health first aid in the design but focused on the workplace environment, addressing common workplace mental health issues such as work-related stress and burnout [6]. The underpinning educational paradigm or approach of the proposed psychoeducation intervention program is called the spaced learning or spaced education approach. Based on the initial neuropsychological concept proposed by the internationally acclaimed neuroscientist Fields [7] and modified and advocated by Kelly [8], the spaced learning approach is a learning methodology for creating long-term memories. This concept is operationalized by presenting highly condensed learning materials repeatedly, based on a predesigned temporal pattern and a number of presentations, to allow the individual to encode the material into one’s memory system. The repetition of the process will reinforce the encoding mechanism and, in turn, commit the materials into long-term memory [8]. Applying this empirically validated approach to education and training, the learning materials could be presented repeatedly in a temporally sequential manner to allow the learner to generate long-term memory. In the design of the psychoeducation component of the intervention program, the principle of spaced education was applied, with various modules of web-based and mobile materials presented to the participant in a progressive manner and some simple exercises presented at the end of each module. Successful completion of 1 module gained access to the next progressively until all modules were completed. Within each module, essential materials were presented repeatedly in a short duration with a temporal pattern of 3 times in a row for the participant to create a long-term memory. The spaced learning or spaced education approach has been used in different fields of training and education, such as continuous medical education [9,10]. Different versions of the main interface, as a website for PCs and as a downloadable mobile app for tablets and smartphones, are presented in Figure 1.

mHealth is defined by the World Health Organization (WHO) as technology “used for medical and public health practice supported by mobile devices, such as mobile phones, patient monitoring devices, Personal Digital Assistants (PDAs), and other wireless devices” [11]. With the rapid development of mobile technologies and further advancement in telecommunication platforms in recent years, mHealth has been adopted as one of the widely used approaches in preventive medicine across different diseases [12-16]. For example, mHealth was used for the promotion of a healthy lifestyle in the prevention of cardiovascular diseases and diabetes [13,16], sexually transmittable infections [9], and cancer [8]. In the prevention of mental health problems, the mHealth approach was also adopted for alcohol and other substance abuse, suicide prevention, and other problems [17-20].

In terms of the effectiveness of the mHealth approach on mental health problems, review studies found some evidence for a positive effect on the reduction of risk factors, such as a reduction in depressive symptoms and stress and an increase in coping, but not on the actual behavior [17,18]. This might be due to the small number of studies included in the reviews since mHealth is still a relatively novel approach. There is also a concern that a number of the studies under review had methodological issues or a lack of proper evaluation that might have contributed to the nonsignificant results obtained [21]. As such, it was recommended that more methodologically robust research should be implemented to further ascertain the efficacy of intervention programs using the mHealth approach [21].

To evaluate the efficacy of the mHealth web-based and mobile WPMHL intervention program, a wait-listed cluster randomized controlled trial (CRCT) was conducted, with MHL as the primary outcome and work-related stress and burnout as the secondary outcomes. The immediate effect of the intervention program was described in a previous report, suggesting a significant result on stress and burnout in favor of the intervention [22]. In terms of MHL, the intervention group scored significantly higher than the control group in the correct recognition of mental problems, help seeking, and stigmatization by 0.2 (SE 0.1; *P*=.003), 0.9 (SE 0.2; *P*<.001), and 1.8 (SE 0.4; *P*<.001), respectively [22]. These results provided evidence for the immediate effect of the psychoeducation component of the program in improving the mental health of the participants [22].

In this study, we aim to focus on both the immediate and 3-month medium-term effects of the intervention program on the MHL of workers. We also aim to report the results obtained for the evaluation of the mHealth psychoeducation platform, particularly engagement, functionality, aesthetics, and the information provided. It is anticipated that the psychoeducation modules will be efficacious in improving MHL immediately and that the effect will remain in the medium term.

## Methods

### Study Design and Target Population

As aforementioned, the protocol for this phase-III, wait-listed CRCT targeting the workplace was reported previously [6]. In brief, 6 large corporations were invited to participate in the trial, and employees were recruited through their corresponding human resources departments. Voluntary participation of recruits was ensured without any influence from the companies’ management. The recruited sample consisted of a wide range of work natures, ranging from manual labor to senior executives, since the business of these corporations covered a multitude of work types. The trial was conducted between March and December 2021 with a 3-month follow-up phase in both intervention and control arms (Checklist 1).

### Recruitment of Participants and Randomization

The primary unit of randomization was the different offices or units of the participating corporations, and recruited workers were clustered in each office or unit. Potential participants were screened for their eligibility at the point of recruitment. Workers who had been exposed to any similar psychoeducation training were excluded from the trial. To randomly allocate the offices and units, the human resources departments of these corporations provided a list of participating offices and units with some basic staffing information, such as the number of staff in the offices and units and their positions. A qualified statistician, who was blinded to the process of recruitment and the subsequent operation and data collection, conducted the randomization using a block randomization process. Each cluster with participants nested in different offices or units was then allocated to either the intervention or wait-listed control groups. Once the cluster had been randomized, participants completed the baseline data collection using the built-in feature of the web-based platform. Postintervention data collection took place immediately after the completion of the psychoeducation modules and at the 3-month follow-up.

### Intervention and the Platform

Details of the intervention program were described in the *Introduction* section. Regarding the mHealth platform, as aforementioned, it was specifically designed for this project by a software company. It was developed to be adaptable for multiple interfaces with web (PC) and app (tablet and smartphone) versions with identical content and functionalities. For the study, participants were provided with a unique log-in to a central server for accessing the platform upon random allocation. Participants who were allocated to the wait-listed control group could activate their log-in when the group changed from the control phase to the intervention phase. Participants could access the web-based platform directly through the server, or they could download the mobile app from the server to be used on their tablets or smartphones for both Android and iOS.

### Study Outcome and Outcome Measures

For the outcome measures of this study, namely MHL and the evaluation of the mHealth platform, 2 instruments were used. For MHL, the Australian National MHL and Stigma Survey was used [23]. The instrument has been validated and widely used in many studies in different countries [24]. In this study, the full survey was not used, given that it is a lengthy instrument and comprises many different submodules, with each module adopting a validated scale that can be used separately. After considering the specific local context, some components of the instrument were selected. These included the correct identification of mental health problems, help seeking for a mental health problem, stigmatization, and social distancing. The responses of these scales were set in a positive direction such that a higher score represented a higher level of the construct. Therefore, a lower score on the stigmatization and social distancing scales reflected a lower level in both constructs. In terms of the evaluation of the mHealth platform, the Mobile App Rating Scale (MARS) was used to assess the quality of different aspects of the platform, including engagement, functionality, aesthetics, and the information provided [25]. The MARS is an instrument designed to assess the quality of mHealth apps developed for addressing health problems or improving health status [25]. As such, it includes an app-specific domain for assessing specific health literacy. It has been validated with a good internal consistency with a Cronbach α value of .90 and a moderately high interrater reliability with an intraclass correlation coefficient of 0.79 [25]. It has also been widely used for evaluating mHealth apps worldwide and translated into different languages [26,27]. Both the MARS and MHL assessments were self-reported, and data were collected through a web-based self-reported survey at different time points. Participants’ information was also collected. This included demographics; employment status; and health behaviors, such as drinking, smoking, and physical activity.

### Procedures

As per the protocol of a wait-listed CRCT, participants in the control arm were offered the same intervention upon the completion of the intervention program by the intervention arm. Hence, all participants received the same intervention treatment eventually. As a result, data collection on MHL was planned to be conducted at 3 different time points: at baseline, immediately after the completion of the program, and 3 months after the completion of the program. Participants were invited to respond to the data collection survey 3 months after the completion of the intervention program with multiple waves of invitations through emails. For the evaluation of the app, a small group of raters was recruited from a different population of workers who were not involved in the trial. They were invited to use the app and then evaluate it using the web-based questionnaire that included the MARS. Training was provided to these raters prior to their exposure to the platform. The raters were invited to rate the platform from a user’s perspective in order to mimic the user’s experience.

### Sample Size and Data Analysis

Based on the assumed effect size of an approximate 0.5 SD difference in the MHL scores between the intervention and control arms, 80% power of the study, a type I error rate of 5%, and an interclass correlation of 0.01, it was estimated that about 400 workers were required. It was also assumed that 10% of participants would drop out of the project. Data were analyzed using the statistical software program Stata BE18.0 (StataCorp LLC). Data obtained from the MARS were analyzed descriptively, with means and SDs or SEs calculated. As suggested by the authors of the scale, the rating was designed as a 5-point Likert scale with 1=inadequate, 2=poor, 3=acceptable, 4=good, and 5=excellent [25]. The mean score of each aspect of the platform was interpreted following the suggested levels of quality and specific health literacy. For MHL, the analysis focused on the efficacy of the intervention program and the changes in outcome measures across time. For investigating the efficacy of the intervention program, comparisons of the mean scores of the outcome measures between the intervention and control groups after the intervention program, adjusting for the clustering effect, and the baseline assessment of the outcome measures were conducted. Given that participants were nested in different clusters and the outcome variables were measured repeatedly, these factors were taken into consideration for the analyses. As a result, a generalized linear latent and mixed model was applied to test the time effect while adjusting for the clustering effect. To handle any loss to follow-up, the main analyses were conducted according to the intention-to-treat principle, and missing data in any variables were imputed using the multiple imputation approach with an assumption of missing at random for all missing values. Since all respondents should have received the intervention at follow-up, including those in the control group, the focus of the comparison was mainly on the change of MHL scores across time with adjustment for the clustering effect. A type I error rate of 5% was adopted for the testing of hypotheses.

### Ethical Considerations

Participation in the trial was voluntary, and no compensation was provided. Potential participants were provided with information on the trial before participation through the corporations’ human resource departments. Willing participants were enrolled in the trial through direct, personal contact with the research team. Informed consent was implied when the participant logged in to the web-based platform. All participants were free to opt out at any time. Confidentiality and privacy were ensured with minimal personal information collected for enrollment purposes, and this information was stored separately on a password-protected database from the trial data. All data collected from the trial were deidentified and stored on a double-layered, password-protected database. The study obtained human ethics approval from the Human Research Ethics Committee of the Tung Wah College (approval REC2018020). Trial registration was also completed with the Australian New Zealand Clinical Trials Registry (ANZCTR; registration ACTRN12619000464167).

## Results

### Primary Outcome of MHL

The results obtained from the wait-listed CRCT, including the demographic and other health-related information of the full sample, had been reported previously [22]. Interested readers should refer to the results in the published paper. In brief, the mean age of the 456 participants was 40.7 (SD 9.8) years, with 215 (47.2%) being male and the majority (n=363, 79.6%) having attained an education level of university or above. Slightly more than half (n=271, 59.4%) were married, and nearly all (n=454, 99.6%) worked full-time. In terms of their health, the majority (n=344, 76.6%) did not exercise regularly; however, very few were reported to be regular smokers (n=17, 3.6%) and drinkers (n=8, 1.8%). A total of 229 (50.2%) participants were randomized to receive the intervention program first, and the rest (n=227, 49.8%) were wait-listed controls providing data for the analyses for the immediate effect. As reported previously, comparisons of the demographics and health-related variables at baseline between groups found no significant differences at all (all *P*>.05). The results obtained on the immediate effect of the psychoeducation program on MHL are summarized in Table 1. As shown, significant differences between groups were observed in all domains except social distancing. After adjusting for the clustering effect and the potential confounding factors, the postintervention weighted mean scores were significantly higher in the intervention group than the control group for correct recognition of a mental health problem, help seeking, and stigmatization by 0.2 (SE 0.1; *P*=.003), 0.9 (SE 0.2; *P*<.001), and 1.8 (SE 0.4; *P*<.001), respectively.

Of the 456 participants, 236 (51.8%) responded to the follow-up survey after multiple waves of invitations and reminders. Comparisons of the demographic variables between respondents and nonrespondents of the follow-up survey indicated no significant differences in basic demographics (all *P*<.05). The demographics and health-related variables of the respondents are summarized in Table 2.

**Table 1.** Different aspects of mental health literacy (MHL) assessed immediately after the intervention by groups and the results on comparisons between groups (N=456).

MHL domains	Intervention (n=229), mean (SE)	Control (n=227), mean (SE)	*P* value^a^
Correct recognition	3.4 (0.1)	3.2 (0.1)	.003
Help seeking	12.9 (0.3)	11.9 (0.2)	<.001
Stigmatization	26.3 (0.5)	24.5 (0.6)	<.001
Social distancing	11.9 (0.5)	12.3 (0.6)	.16

a Adjusted for the clustering effect, age, education level, and baseline assessment of the outcome measure.

**Table 2.** Demographics and health-related variables of respondents who responded at baseline, postintervention, and 3-month follow-up (n=236).

Characteristics		Value^a^
**Demographics**		
	Age (years), mean (SD)	42.8 (9.7)
	Male sex, n (%)	107 (45.3)
	Education level (university or above), n (%)	184 (78)
	Marital status (married), n (%)	155 (65.7)
	Full-time employment (yes), n (%)	236 (100)
**Health-related variables, n (%)**		
	Regular exercise (yes)	168 (71.2)
	Smoker (yes)	6 (2.5)
	Drinker (moderate or heavy)	4 (1.7)

a Adjusted for the clustering effect.

The results of the changes in the MHL outcome measures and the comparisons are presented in Table 3. As shown, of the 4 main MHL outcomes, all but 1 yielded a significant time effect. After adjusting for the clustering effect, significant differences across time were found in help-seeking intention (*P*=.01), stigmatization (*P*<.001), and social distancing (*P*<.001). As suggested from the mean values, on the whole, there was an increase in help-seeking intention scores and a reduction in stigmatization and social distancing scores over time.

**Table 3.** Different aspects of mental health literacy (MHL) at baseline, postintervention, and 3-month follow-up and the results on the time effect (n=236).

MHL domains	Baseline, mean (SD)	Postintervention, mean (SD)	3-month follow-up, mean (SD)	*P* value
Correct recognition	3.2 (0.6)	3.4 (0.7)	3.3 (0.7)	.20
Help seeking	12.4 (1.9)	12.7 (2.3)	12.7 (2.1)	.01
Stigmatization	24.7 (3.6)	25.8 (4.1)	25.8 (3.9)	<.001
Social distancing	12.5 (3.2)	12.1 (3.3)	11.9 (3.5)	<.001

### Evaluation of the mHealth Platform

For the evaluation of the mHealth Platform, a total of 70 individuals were recruited. Of these, there were 26 (37%) male individuals with an average age of 34.2 (SD 1.6) years. None of these participants had been exposed to any similar psychoeducation materials or mHealth platforms prior to the evaluation exercise. The results of the evaluation are summarized in Table 4. As shown, the average scores of the 4 major domains ranged from 3.8 to 4.2, with engagement having the lowest score. For the perceived impact on MHL, all items were scored higher than 4.0, suggesting a greater tendency of respondents to endorse the positive impact of the content on their MHL.

**Table 4.** Different domains of the Mobile Application Rating Scale (MARS) and the perceived impact on mental health literacy (n=70).

Variable		Value, mean (SD)
**MARS domains**		
	Engagement	3.8 (0.6)
	Functionality	4.2 (0.5)
	Aesthetics	4.0 (0.5)
	Information	4.2 (0.5)
	Overall	4.0 (0.4)
**Perceived impact on mental health literacy**		
	Awareness	4.4 (0.6)
	Knowledge	4.4 (0.5)
	Attitude	4.4 (0.6)
	Intention to change	4.3 (0.6)
	Help seeking	4.4 (0.6)
	Behavior change	4.3 (0.6)

## Discussions

### Principal Findings

This study was a continuation of a wait-listed CRCT of the effect of a psychoeducation intervention program using an mHealth spaced education approach on workplace MHL. The main results of the immediate efficacy of the intervention program had been reported previously [22]. This study aimed to further report the results obtained on the evaluation of the mHealth platform and the medium-term effect of the intervention program on the MHL of participants. Based on the MARS scores of different aspects of the platform, the results have demonstrated that the mobile platform is well acceptable, with an overall score of 4.0 (SD 0.4) and individual domain scores ranging between 3.8 and 4.2. This represents a generally good reception of the platform by the respondents in terms of the functionality, aesthetics, and the information provided. The result for the engagement domain was slightly below the level of good, in accordance with the classification of the original author of the MARS [25]. These results are compatible with the results obtained from a study rating 50 mental health and well-being mobile apps using the MARS by the original authors [25]. The average domain scores of these 50 apps ranged between 2.7 to 4.0 [25]. In comparison, the mHealth platform has shown better performance than these reported results.

The immediate effect of the psychoeducation intervention program on MHL was interpreted and discussed previously [22]. The significant and positive results indicated the efficacy of the intervention program in improving workers’ MHL. There were significant improvements in 3 of the 4 main domains of MHL, including correct recognition, help seeking, and stigmatization, after the intervention. In terms of the medium-term sustainability of these gained benefits, the results obtained from the follow-up phase of the study provided some evidence. As shown, these effects were sustained 3 months after the intervention, with further improvement in social distancing, as reflected in the average scores. These results suggest the retention of the gained benefits in the enhancement of MHL in these participants. These results are consistent with those in the literature. A recent systematic review and meta-analytical study on the long-term effects of interventions for MHL in children and adolescents reported similar results [28]. In pooling data from 25 studies with an average follow-up period of 5 months, it was found that the improvement in MHL was sustained, particularly for stigmatization (*d*=0.30, 95% CI 0.24-0.36) and social distancing (*d*=0.16, 95% CI 0.03-0.29) [28]. When combining the results obtained from the evaluation exercise using the MARS, the results provide stronger evidence that the mHealth psychoeducation intervention program is effective in improving and retaining the gain in the MHL of workers in the workplace.

The *Mental Health Action Plan 2013-2020* produced by the WHO has affirmed the importance of the workplace as a venue for mental health education and advancement [2]. The results reported previously by the authors, in conjunction with those obtained from this study, provide further evidence that a well-developed and executed psychoeducation intervention program in the workplace can not only reduce burnout and stress but also enhance MHL. Moreover, the effect of MHL enhancement can be sustained and, to a certain extent, further improved over time. The results of this study have also demonstrated that a well-designed and engaging mHealth platform with good functionality and aesthetics would also be a vehicle for enhancing MHL. As aforementioned, the mHealth approach for disease treatment, management, and health promotion has been used in many different health areas [11,12]. However, it is still considered a rather recent area of development according to some scholars in the field [15]. It is anticipated that the COVID-19 pandemic could be a catalyst in stimulating and motivating health professionals to develop and adopt digital health more readily and rapidly [29]. With the latest development in the field of data science and artificial intelligence, it is also expected that greater health advancements could be achieved through personalized information provision for education and promotion [30].

The strengths and limitations of the wait-listed CRCT were discussed in the previous report and will not be reiterated [22]. In this study, the focuses were the evaluation of the mHealth platform and the medium-term effect of the intervention program. One of the strengths of the study is the use of standardized and validated assessment instruments for outcome variables. The MARS and MHL measures are widely used instruments with ample evidence for their reliability and validity. As a result, the measurement and interpretation biases can be minimized. Another strength of the study is the samples recruited for the evaluation study and the CRCT. The random sample of the CRCT consisted of employees of different large-sized industries, thus covering a wide range of work nature and seniority. Hence, it could be considered a representative sample. For the evaluation study, the sample also consisted of participants from different companies and working backgrounds, thus preserving some degree of representation of the target working population. A few limitations have been identified in this study. The design of the mHealth platform for the psychoeducation intervention program is based on the spaced education concept. However, in the evaluation of the platform, the use of the MARS could only focus on the design aspects of the app but not the effect of the approach on participants’ learning. As a result, the effect of the spaced education approach adopted in this platform could not be evaluated. Another drawback of the study on the medium-term effect of the program is the low follow-up rate, with slightly more than half (236/456, 51.8%) of the original trial sample fully completing all assessments. This may incur a response bias in the follow-up study, although it has been demonstrated that there are no significant differences in the demographic variables between the follow-up and non–follow-up groups. To evaluate the learning effect of the spaced education approach, it is suggested that a randomized controlled trial with the use of an appropriate assessment instrument should be conducted. For minimizing the loss to follow-up, as suggested by some trialists, more frequent contact and better communication could help in retaining trial participants [31].

### Conclusion

In conclusion, the web-based and mobile spaced education psychoeducation intervention program (WPMHL) has immediate and 3-month medium-term effects of retaining and improving MHL. The platform performed satisfactorily in terms of functionality, aesthetics, information content, and utility in enhancing MHL. It is anticipated that ongoing development in digital health will provide great benefits in improving the mental health of the global population.

## Supplementary material

10.2196/51791 Checklist 1 CONSORT-eHEALTH checklist (V 1.6.1).

## Abbreviations

ANZCTR: Australian New Zealand Clinical Trials Registry
CRCT: cluster randomized controlled trial
MARS: Mobile Apps Rating Scale
mHealth: mobile health
MHL: mental health literacy
WHO: World Health Organization
WPMHL: Workplace Mental Health Literacy
